# The Association Between Epigenetic Clocks and Physical Functioning in Older Women: A 3-Year Follow-up

**DOI:** 10.1093/gerona/glab270

**Published:** 2021-09-20

**Authors:** Tiina Föhr, Timo Törmäkangas, Hannamari Lankila, Anne Viljanen, Taina Rantanen, Miina Ollikainen, Jaakko Kaprio, Elina Sillanpää

**Affiliations:** Faculty of Sport and Health Sciences, Gerontology Research Center (GEREC), University of Jyväskylä, Jyväskylä, Finland; Faculty of Sport and Health Sciences, Gerontology Research Center (GEREC), University of Jyväskylä, Jyväskylä, Finland; Faculty of Sport and Health Sciences, Gerontology Research Center (GEREC), University of Jyväskylä, Jyväskylä, Finland; Faculty of Sport and Health Sciences, Gerontology Research Center (GEREC), University of Jyväskylä, Jyväskylä, Finland; Faculty of Sport and Health Sciences, Gerontology Research Center (GEREC), University of Jyväskylä, Jyväskylä, Finland; Institute for Molecular Medicine Finland (FIMM), University of Helsinki, Helsinki, Finland; Department of Public Health, University of Helsinki, Helsinki, Finland; Institute for Molecular Medicine Finland (FIMM), University of Helsinki, Helsinki, Finland; Faculty of Sport and Health Sciences, Gerontology Research Center (GEREC), University of Jyväskylä, Jyväskylä, Finland; Institute for Molecular Medicine Finland (FIMM), University of Helsinki, Helsinki, Finland

**Keywords:** Biological aging, DNA methylation, Epigenetic clock, Physical functioning

## Abstract

**Background:**

Epigenetic clocks are composite markers developed to predict chronological age or mortality risk from DNA methylation (DNAm) data. The present study investigated the associations between 4 epigenetic clocks (Horvath’s and Hannum’s DNAmAge and DNAm GrimAge and PhenoAge) and physical functioning during a 3-year follow-up.

**Method:**

We studied 63- to 76-year-old women (*N* = 413) from the Finnish Twin Study on Aging. DNAm was measured from blood samples at baseline. Age acceleration (AgeAccel), that is, discrepancy between chronological age and DNAm age, was determined as residuals from linear model. Physical functioning was assessed under standardized laboratory conditions at baseline and at follow-up. A cross-sectional analysis was performed with path models, and a longitudinal analysis was conducted with repeated measures linear models. A nonrandom missing data analysis was performed.

**Results:**

In comparison to the other clocks, GrimAgeAccel was more strongly associated with physical functioning. At baseline, GrimAgeAccel was associated with lower performance in the Timed Up and Go (TUG) test and the 6-minute walk test. At follow-up, significant associations were observed between GrimAgeAccel and lowered performance in the TUG, 6-minute and 10-m walk tests, and knee extension and ankle plantar flexion strength tests.

**Conclusions:**

The DNAm GrimAge, a novel estimate of biological aging, associated with decline in physical functioning over the 3-year follow-up in older women. However, associations between chronological age and physical function phenotypes followed similar pattern. Current epigenetic clocks do not provide strong benefits in predicting the decline of physical functioning at least during a rather short follow-up period and restricted age range.

Life expectancy, which reflects the health of a population, has increased dramatically over the last decades. Although there are some concerns in developed countries that this might not be the case in the future, globally, the positive trend in life expectancy is expected to continue. Current aging generations are healthier and perform better in several functional tasks than previous ones (1). However, the question remains whether the extended years are spent suffering from common old-age disabilities or whether the functional capacity of older people can enable independent living and promote a good quality of life. Aging is accompanied with physiological changes that lead to declining strength, balance, and gait control. These changes are predisposed to impairments in daily mobility tasks. In particular, balance and gait disorders represent major public health concerns due to their association with falls and fall-related injuries (2). Therefore, developing methods to recognize and rehabilitate individuals who are at high risk of age-related decline in physical functioning would help promote independent living among older people.

Chronological age is not an unambiguous measure of the individual aging process or a predictor of future disability risk. The rate at which an individual ages biologically varies relative to her/his chronological age, as does their risk of age-related decline in physical functioning and disease (3). Measures of mechanisms that influence the biological aging process, health, and functioning may help in identifying people who are at the highest risk as well as in developing targeted therapies. Biological aging is associated with progressive loss of function at the cellular, tissue, and organ levels, further promoting the general decline in physical functioning and cognitive performance (4). For instance, genomic instability, telomere attrition, cellular senescence, epigenetic processes, loss of protein homeostasis (proteostasis), dysregulated nutrient sensing, mitochondrial dysfunction, altered intercellular communication, and stem cell exhaustion are central processes linked to biological aging and aging-related changes in physical functioning such as muscle weakness and reduced neurogenesis (4).

Of these central mechanisms of biological aging, epigenetic processes are reversible chemical and structural alterations to the genome that can lead to long-term changes in gene activity, consequently altering a biological trait or process phenotype without altering the underlying DNA sequence itself (5). DNA methylation (DNAm), attachment of a methyl group to C-5 of the cytosine base in the context of cytosine–phosphate–guanine (CpG) dinucleotide in a DNA strand, is the most commonly studied epigenetic modification. Studies have found evidence of age-related hypo- or hyper-methylation within specific CpG sites or islands (5). These findings have laid the groundwork for the development of epigenetic biomarkers of aging, also referred to as epigenetic clocks. The first 2 widely used epigenetic clocks, Horvath’s DNAmAge and Hannum’s clock, were developed in 2013 to predict chronological age. Horvath’s DNAmAge is based on DNAm at 353 specific CpG sites (6). It has been argued that these “first-generation clocks” may exclude CpGs whose methylation patterns may reflect a variation in the biological age, because the algorithms were trained against chronological age (7). In recent years, new DNAm-based biomarkers for aging have been developed, capturing CpGs associated with the functional stage along with chronological age. Levine’s PhenoAge is a predictor of phenotypic age, while the DNAm GrimAge clock was developed to predict life span (8). GrimAge is a linear combination of DNAm-based surrogate biomarkers for health-related plasma proteins, smoking pack-years, sex, and chronological age (8). It is a stronger predictor of age-related conditions, disease, and mortality risk compared to the widely used Horvath’s DNAmAge (9,10).

The association between epigenetic clocks and physical functioning and the ability of clocks to predict the future development of disabilities is largely unknown, though highly interesting. Previous evidence from a small number of longitudinal studies (11–13) have been inconsistent and have focused on “first-generation” clocks. Additionally, the range of physical performance measures has been quite narrow. In general, performance-based measures in laboratory conditions provide more explicit and standardized information on physical functioning compared to self-assessments. Walking speed and muscle strength tests are widely used performance-based tests of physical functioning. They capture current and preceding lifetime influences on functioning and predict disability and mortality risk (14,15).

The purpose of the present study was to investigate the association between epigenetic aging and physical functioning in 63- to 76-year-old women during a 3-year follow-up. More specifically, we studied associations between 2 epigenetic clocks (Horvath’s DNAmAge and DNAm GrimAge) and measures of physical functioning conducted in standardized laboratory conditions. All analyses were also replicated using Hannum’s DNAmAge and DNAm PhenoAge.

## Method

### Participants and Study Design

The participants were from The Finnish Twin Study on Aging (FITSA), which investigated genetic and environmental effects on the disablement process in older female twins. Participants from the FITSA study were recruited from the Older Finnish Twin Cohort, which comprised all same‐sex twin pairs born before 1958 and both co‐twins alive in 1975 (16). The recruitment process of the FITSA study has been described in detail elsewhere (17,18). Briefly, an invitation to participate in the study was sent to 414 female twin pairs aged 63–76 years. The final sample of the FITSA study included 114 dizygotic (DZ) and 103 monozygotic (MZ) twin pairs (434 individuals), and zygosity was determined from DNA samples. Before the laboratory examinations, the subjects were informed about the study, and written consent was obtained. The FITSA study was approved by the Committee on Ethics of the Central Hospital of Central Finland. The present study utilizes the baseline at 2000–2001 and 3-year follow-up measurements of the FITSA study. The participants with available DNAm age were included in the present study (*n* = 413).

### DNAm Age Acceleration

The generation, preprocessing, and normalization of the DNAm data were described in our previous paper (19). Briefly, genome-wide DNAm from blood samples was determined on Illumina EPIC BeadChip, and the data were preprocessed with R package *minfi*. Detection *p* values comparing the total signal for each probe to the background signal level were calculated to evaluate the quality of the samples (20). Further analysis excluded samples of poor quality (mean detection *p* > .01). The single-sample Noob normalization method was used to normalize the data (21). The epigenetic age estimates, including Horvath’s (6) and Hannum’s (22) DNAmAge, DNAm PhenoAge (7), and GrimAge (8), were produced using an online calculator with default settings (https://dnamage.genetics.ucla.edu/new). Epigenetic age acceleration, which describes the difference between the chronological age and the epigenetic age estimate, was calculated as the residual from a linear regression model of epigenetic age estimates of chronological age. DNA collection and the assessment of DNAm age acceleration were conducted only at baseline.

### Laboratory Measurements

The participants took part in laboratory measurements at baseline and at the 3-year follow-up. Before the functional performance measurements, their health status, chronic conditions, and medication were carefully evaluated by a physician during a 30-minute clinical examination. Smoking status (current, former, or never) and alcohol use were assessed by a standardized questionnaire. Use of alcohol was measured as beverage type-specific items on frequency and quantity and converted into grams of absolute ethanol per day. Chronic diseases were first self-reported and then confirmed during the medical examination. Chronic diseases considered here included chronic cardiovascular, pulmonary, neurological, musculoskeletal, and metabolic diseases as well as all cancers (23). Number of chronic diseases was calculated by adding up the diagnoses. Attention was paid to risk factors and contraindications for the measurements. Standardized laboratory measurements of physical functioning, including *the Timed Up and Go (TUG) test*, *the 10-m walk test*, *the 6-minute walk test*, and the *isometric muscle strength tests of grip strength, ankle plantar flexion strength, and knee extension strength*, were undertaken by trained staff, mainly physiotherapists. More detailed information about these tests is provided as Supplementary Material. The participants’ body mass index (BMI) was calculated from measured body weight and height (kg/m^2^).

### Statistics

Descriptive statistics were analyzed using the IBM SPSS Statistics 26 software. The data are shown as means and standard deviations unless otherwise stated. Correlation coefficients and paired samples *t* test were computed to estimate the level of similarity between chronological age and DNAm age estimates. In addition, Bland–Altman plot, intraclass correlation, and concordance correlation were used to assess agreement of chronological age and DNAm age. Associations of DNAm age acceleration estimates and chronological age with physical functioning at baseline and follow-up were analyzed with the MPlus statistical software (24). The cross-sectional analysis was based on a path model with an unstructured within-twin pair outcome correlation matrix and an adjustment for the within-pair dependence of twin pairs. The longitudinal analysis was performed as a repeated measures linear path model, which included an unstructured outcome correlation matrix due to additional longitudinal within-person dependence. The advantage of this model over potential alternatives was the possibility to account for nonrandom missing data patterns in a flexible modeling framework, which is important in the analysis of a sample of older participants, where the assumption of data missing at random can lead to biased results. We estimated the regression coefficients between epigenetic age acceleration measures and the response variables, assuming that the data were missing not at random (25) in a selection model (26) where factors related to attrition were indicators of missingness regressed on the outcome variables (27). We used the false discovery rate (FDR) to correct the *p* values for multiple testing (28). After correction, *p* values below .05 were considered statistically significant. Further, as additional supplementary analysis, we used several models to investigate impact from adjusting for potential covariates. All models were first adjusted with smoking. Information about the participants’ smoking status (current/former or never) was included in the models as dummy variables. Second set of adjusting factors included number of chronic diseases and alcohol usage in grams. Results from these adjusted models are presented in Supplementary Table 2. Originally, PhenoAge and Hannums’ clocks were external to our study plan, and they were suggested by reviewers. Hence, the association of these age acceleration estimates and physical functioning are shown in Supplementary Tables 1 and 3. Finally, based on preliminary visualization, the relationship between BMI and some of the age acceleration variables seemed to follow a curvilinear pattern, and we decided to model the impact of age accelerations also using quadratic functional form (Supplementary Table 4). Curvilinear associations were modeled using second-degree polynomial: h + h2, where h is the linear component and h2 is the quadratic component.

## Results

### Characteristics

The participants’ baseline characteristics are presented in Table 1. The mean age of the participants was 68.6 years. The mean difference in chronological age and Horvath’s DNAmAge was −1.7 years (*p* < .001) and with DNAm GrimAge −8.7 years (*p* < .001) (Table 1). The correlation coefficient between chronological age and DNAm GrimAge was 0.68 (*p* < .001) and between chronological age and Horvath’s DNAmAge 0.60 (*p* < .001). Agreement assessment between chronological age and DNAmAge measures shown in the supplement suggested that estimates of Horvath’s DNAmAge were on average more similar to chronological age than DNAm GrimAge. The participants were mainly close to normal weight (BMI 27.9 ± 4.7). Of the 413 women with baseline data, 8 (1.9%) died before the laboratory measurements. In total, 298 women (72.2% of the baseline participants, mean age at follow-up 71.3 years) participated in the laboratory measurements at the 3-year follow-up. Three participants were unable to complete the functional measurements.

**Table 1.** Characteristics of the Study Participants (all women) and Physical Functioning at Baseline and After the 3-Year Follow-up

	Baseline	N	3-y Follow-up	N
Age (y)	68.6 (3.4)	413	71.3 (3.3)	298
Epigenetic clocks				
Horvath’s DNAmAge (y)	66.9 (5.7)	413		
Hannum’s DNAmAge (y)	56.4 (6.0)	413		
DNAm PhenoAge (y)	55.1 (7.9)	413		
DNAm GrimAge (y)	59.9 (4.4)	413		
Body mass index (kg/m^2^)	27.9 (4.7)	413	28.1 (5.0)	298
Cigarette smoking				
Current (%)	4.9	20		
Former (%)	7.3	30		
Never (%)	87.9	362		
Physical functioning tests				
Timed Up and Go (s)	9.3 (1.9)	405	9.0 (2.0)	280
10-m walk (s)	6.0 (1.3)	397	5.9 (1.7)	281
6-min walk (m)	531.6 (76.4)	339	528.2 (79.4)	201
Grip strength (N)	190.0 (57.1)	413	191.3 (61.2)	293
Ankle plantar flexion strength (N)	218.4 (84.3)	378	212.4 (80.3)	279
Knee extension strength (N)	293.5 (84.0)	387	282.8 (80.5)	272
Selected diseases				
Coronary heart disease	51 (12.3)			
Heart failure	21 (5.1)			
Hypertension	152 (36.8)			
Asthma	33 (8.0)			
COPD	3 (0.7)			
Rheumatoid arthritis	17 (4.1)			
Hypothyroidism	27 (6.5)			
Hyperthyroidism	11 (2.7)			
Type 2 diabetes	24 (5.8)			
Cancer	35 (8.5)			

*Notes*: COPD = chronic obstructive lung disease; DNAm = DNA methylation. Values are mean (*SD*) unless stated.

### Zygosity Effects

As individuals, the MZ and DZ twins did not differ in the association of epigenetic age acceleration with physical functioning phenotypes at the baseline or follow-up (minimum adjusted *p* = .990); thus, both types of twins were analyzed together.

### Associations Between Epigenetic Age Acceleration and Physical Functioning at Baseline

For consistency, all *p* values below are reported after the FDR correction. In unadjusted models, higher GrimAgeAccel was associated with more time (s) in the TUG test (unadjusted regression coefficient estimate [est.] 0.004, *SE* 0.001, *p* = .003) and a shorter walking distance (m) in the 6-minute walk test (est. −0.004, *SE* 0.002, *p* = .014). Horvath AgeAccel, Hannum AgeAccel, and PhenoAgeAccel were not associated with the TUG, 10-m, or 6-minute walk tests or the isometric strength tests (Table 2; Supplementary Table 1). Associations between GrimAgeAccel and physical functioning remained significant after adjusting for smoking, alcohol usage, and chronic diseases (Supplementary Table 2).

**Table 2.** Unadjusted Regression Coefficient Estimates, Standard Errors, Uncorrected *p* Values and *q* Values for Linear Associations Between GrimAge and Horvath Age Acceleration, and Body Mass Index and Physical Functioning Phenotypes at Baseline (*N* = 413) and at 3-Year Follow-up (*N* = 298) Among Older Women

Outcome	AgeAccel	Time Point	Multiplier	Est.	SE	p	q
Body mass index (kg/m^2^)	GrimAge	Baseline	—	0.036	0.085	.674	.809
		Follow-up	—	0.022	0.090	.804	.889
	Horvath	Baseline	—	0.101	0.049	.040	.093
		Follow-up	—	0.085	0.053	.108	.174
Timed up and go (s)	GrimAge	Baseline	10	0.004	0.001	.001	.003
		Follow-up	10	0.005	0.002	.003	.009
	Horvath	Baseline	10	0.000	0.001	.734	.856
		Follow-up	10	0.000	0.001	.995	.995
10-m walking test (s)	GrimAge	Baseline	—	0.001	0.001	.054	.108
		Follow-up	—	0.003	0.001	<.001	.002
	Horvath	Baseline	—	0.000	0.000	.935	.982
		Follow-up	—	0.001	0.000	.234	.339
6-min walking test (m)	GrimAge	Baseline	1/1 000	−0.004	0.002	.005	.014
		Follow-up	1/1 000	−0.006	0.002	.001	.003
	Horvath	Baseline	1/1 000	0.000	0.001	.934	.982
		Follow-up	1/1 000	−0.001	0.001	.355	.481
Grip strength (N)	GrimAge	Baseline	1/100	−0.010	0.008	.208	.312
		Follow-up	1/100	−0.015	0.010	.137	.213
	Horvath	Baseline	1/100	−0.002	0.006	.760	.863
		Follow-up	1/100	0.013	0.007	.054	.108
Ankle plantar flexion strength (N)	GrimAge	Baseline	—	−0.116	0.066	.079	.133
		Follow-up	—	−0.217	0.082	.008	.021
	Horvath	Baseline	—	−0.044	0.056	.433	.568
		Follow-up	—	0.035	0.059	.557	.709
Knee extension strength (N)	GrimAge	Baseline	1/100	−0.032	0.017	.059	.113
		Follow-up	1/100	−0.053	0.016	.001	.003
	Horvath	Baseline	1/100	0.024	0.013	.077	.133
		Follow-up	1/100	0.012	0.012	.317	.444

*Notes*: AgeAccel = DNA methylation age acceleration; Est. = unadjusted regression coefficient estimate; *p* = expected false positive rate for a single test; *q* = expected false positive discovery rate among all outcomes; *SE* = standard error based on 10 000 bootstrap draws. Outcome values were scaled with the multiplier value. A nonrandom missing data mechanism was utilized in longitudinal models.

### Associations Between Epigenetic Age Acceleration and Physical Functioning at the 3-Year Follow-up

The longitudinal analysis indicated that GrimAgeAccel was associated with declined performance in the TUG test (est. 0.005, *SE* 0.002, *p* = .009) and 10-m (est. 0.003, *SE* 0.002, *p* = .002) and 6-minute walking tests (est. −0.006, *SE* 0.002, *p* = .003) and with lowered ankle plantar flexion strength (est. −0.217, *SE* 0.082, *p* = .021) and knee extension strength (est. −0.053, *SE* 0.016, *p* = .003) in the unadjusted models. Horvath and Hannum AgeAccel and PhenoAgeAccel were not associated with changes in physical functioning. In adjusted models, association between GrimAgeAccel and decline in ankle plantar flexion strength was attenuated, but other associations remained significant.

### Associations Between Chronological Age and Physical Functioning at Baseline and at the 3-Year Follow-up

In baseline measurements, higher chronological age was associated with worse performance in TUG and walking tests and lower isometric muscle strength in ankle plantar flexion and knee extension (Supplementary Table 1). At follow-up, higher chronological age was associated with a declining performance in TUG, walking tests, and grip and knee extension strength.

### Associations Between Epigenetic Age Acceleration and BMI at Baseline and at the 3-Year Follow-up

In linear models, only higher PhenoAgeAccel was modestly associated with higher BMI at baseline (est. 0.072 *SE* 0.036, *p* = .047) (Table 2; Supplementary Table 1). In quadratic models, GrimAgeAccel was associated with BMI at baseline and at follow-up, suggesting that age deceleration and acceleration were more prominent among lean subjects and closer to zero among those with higher BMI. These associations remained after adjustment for lifestyle factors. PhenoAgeAccel associated with BMI only at baseline and other quadratic associations were not observed.

## Discussion

The present study employed a longitudinal design to investigate associations between markers of biological aging, that is, epigenetic clocks and validated physical functioning phenotypes. Both the “first-generation” clocks and novel “second-generation” clocks were utilized in the analysis. We found that DNAm GrimAge, which was developed to predict life span and healthspan (29,30), associated with a decline in physical functioning, while other clocks (6) showed no associations with physical functioning. More specifically, GrimAgeAccel was associated with lower performance in the TUG test and 6-minute walk test at baseline and with declining performance in the TUG test, 6-minute and 10-m walking tests, and ankle plantar flexion and knee extension strength test during the 3-year follow-up. However, chronological age provided very similar estimates in cross-sectional and longitudinal analyses. In sum, our results suggest that DNAm GrimAge outperformed other epigenetic clocks in predicting age-related decline in physical functioning. Current epigenetics clocks, however, do not provide special benefits in predicting later decline in physical functioning, at least during a rather short follow-up period and narrow age range.

The present study utilized validated laboratory tests to assess participants’ strength, balance, and walking ability. These factors are negatively affected by aging and are important determinants of physical functioning and future disability. For instance, earlier research suggests aging is associated with a progressive decline in muscle strength, which is shaped by genetic background and multiple environmental factors, leading to significant diversity in physical functioning in older age (17,18). Muscle strength peaks at 20–30 years of age and declines slowly thereafter (31). After age 50, muscle strength loss accelerates, and previous studies have shown that poor muscle strength is a risk factor for disability, morbidity, and mortality (32,33) and that higher muscle strength protects against the onset of future disability in older people (34). Maximal isometric strength tests are considered to provide useful information about physical functional capacity in older people (35). The results of the present study showed that higher GrimAgeAccel was associated with a greater decline in ankle plantar flexion and knee extension strength at follow-up.

Aging is associated with a number of structural and functional changes in the cardiovascular system and progressive loss of aerobic performance, which have significant implications for cardiovascular disease and mortality (36). An accurate predictor of aerobic performance is maximal oxygen consumption, measured during a maximal running or cycling test until voluntary exhaustion. This type of testing procedure cannot be used in older people due to apparent health risks. Therefore, submaximal tests that correlate with the results of maximal aerobic tests and are related to functional capacity under normal living conditions are used among older populations. Of these tests, the 10-m walk test for gait speed and the 6-minute walk test for submaximal fitness are commonly used to assess daily activity performance in older people. Our results showed an association between higher GrimAgeAccel and shorter walking distance (m) in the 6-minute walk test and declined performance in both 6-minute and 10-m walking tests at follow-up.

In addition to strength and walking ability, balance is considered an important determinant of physical functioning and independence (17,18). Balance is required for maintaining a static posture, stabilizing dynamic movements, and performing daily activities. Aging and chronic diseases can worsen the balance ability of older people; therefore, an assessment of balance ability can help in predicting and preventing both falls and a reduction in independent living among older people (37). The TUG test is considered an appropriate measure of balance performance for older people (37). Previous studies have found it to be an acceptable tool to predict disability in activities of daily living or instrumental activities of daily living (34,38,39). We found an association between GrimAgeAccel and poorer time in the TUG test at baseline. Further, the results indicated that higher GrimAgeAccel was associated with a decline in physical functioning measured by the TUG test.

To our knowledge, only a few studies have investigated the association between DNAm GrimAge and physical functioning. In line with our findings, cross-sectional analyses have found higher GrimAgeAccel to be associated with slower walking speed but not with a decline in grip strength (40). The longitudinal study by Maddock et al. (11) examined the association between GrimAgeAccel and decline in physical functioning (grip strength, chair rise speed, and lung function) over a 16-year follow-up. They found an association between higher GrimAgeAccel and greater decline in grip strength between 53 and 69 years (11). They also found that high GrimAgeAccel at 53 years was associated with a poorer chair rise speed at 53 and 69 years but not with decline in grip strength. In contrast to the findings of Maddock et al. (11), we did not observe a decline in grip strength during the 3-year follow-up. The differences in the time window and length of the follow-up period may explain the difference between our findings and those of Maddock et al. Another explanation may be that both women and men were included in the study by Maddock et al., but our study only included women.

Among older people, accelerated aging is typically associated with declining physical activity (25), which is also a determinant of physical functioning. First, existing results regarding the associations between GrimAgeAccel and physical activity have been controversial, reporting either no association (41) or an inverse association, suggesting that low levels of physical activity are associated with an accelerated aging pace (42). The conflicting findings arguably relate to differences in the mode of physical activity; thus, previous results from our group suggest that leisure-time physical activity and occupational physical activity have transversal associations with epigenetic aging (19). Different measurement techniques and ways of collecting physical activity data and different age spans may also explain the discrepancy between earlier findings. It has also been hypothesized that associations between epigenetic aging and physical activity may vary by ethnicity (8).

The present study found no cross-sectional or longitudinal associations between age acceleration measured by Horvath’s and Hannum’s DNAmAge, and PhenoAge and multiple measures of physical functioning. Previous studies using Horvath’s DNAmAge as a measure of DNAm age were partly inconsistent but mainly in line with our findings. Accelerated Horvath DNAm age has been found to be associated with both lower (13,43) and higher (40) grip strength. Previous studies found no association between Horvath’s DNAmAge and walking speed (13,40,43) or knee extension strength (43). Previous longitudinal studies found no associations between Horvath’s DNAmAge and grip strength or lung function at 6-year (13) and 16-year (11) follow-ups. However, while Simpkin et al. (12) found accelerated Horvath DNAmAge to be associated with a greater decrease in grip strength, they observed no associations with standing balance time or chair rise speed (12). The newer clock DNAm PhenoAge is based on “PhenoAge,” a separate measure of biological age comprising age and 9 clinical biomarkers (7). Higher PhenoAgeAccel has been reported to be associated with excess adiposity (42) and some age-related clinical conditions such as cancers (44,45), but associations with physical functioning have been reported rarely and results are inconsistent (40,46). Based on our current results, it seems that PhenoAge is not a strong predictor of age-related functional decline.

The number of smokers was exceptionally low among the participants in the present study (former smokers *n* = 30 [7.3%], current smokers *n* = 20 [4.8%]). Nevertheless, we recognized that the smoking status (nonsmoker, former/current smoker) had to be acknowledged in the analyses. Smoking is known to have several adverse effects on human health, including biological aging (47). It is considered one of the most detrimental lifestyle factors and is associated with an increased risk of multiple diseases, including various forms of cancer and respiratory and cardiovascular disease (47,48), accelerated cellular aging (49), and mortality (47,50,51). Active smoking in adults is a critical factor for DNAm, which has been found to play a significant role in the pathways of smoking and smoking-related diseases (47). According to a meta-analysis by Pan et al. (50), smoking was prospectively associated with around a 50% increased risk of cardiovascular events and total mortality. The harmful health-related effects of smoking are often combined with other unhealthy lifestyle factors that typically accumulate among smokers (52). However, the results of a recent study by Fiorito et al. (53) showed that smoking had a stronger effect on epigenetic aging compared to socioeconomic position, obesity, and alcohol intake (53). Additionally, it was important to acknowledge the effect of smoking in the analysis because smoking was taken into account in the development of DNAm GrimAge, whose estimates represent combined information on chronological age, sex, and DNAm-based surrogate biomarkers for 7 plasma proteins and smoking pack-years (8).

Missing data is a serious challenge in aging research. In longitudinal studies, death and loss to follow-up increase with age. In addition, health problems and functional limitations are more common in older age and interfere with all aspects of data collection. Missing data can bias results, reduce generalizability, and limit statistical power. For instance, in our study, 91 (30.5%) of the twin individuals who participated in the follow-up measurements did not obtain permission from a doctor to conduct the 6-minute walk test or did not want to or were unable to complete the test. The corresponding numbers were 15 (5.0%) for the TUG test and 14 (4.7%) for the 10-m walk test. It was clear that the loss of data at follow-up was not due to nonrandom missingness but was caused by factors associated with the biological aging process. Therefore, we used advanced statistical methods to handle missing data resulting from both mortality and decreased physical functioning in our longitudinal research setting. The follow-up period of the present study was relatively short. However, the follow-up period was in a suitable time window with respect to the development of functional limitations, and we were able to observe significant decline in physical functioning during such a short follow-up. This study only included female twin pairs with a rather narrow age span, which limited the generalizability of the results. Nevertheless, the narrow age span of the participants substantiated our findings in this specific phase of life. It also has to be noted that there is a possibility for selection bias. Our study sample included older women of whom very few were smokers (23) and who were willing and able to travel to the laboratory and participate in functional tests. These participants were epigenetically younger compared to their chronological age, for example, in GrimAge, the mean of the individual differences in DNAm age compared to chronological age was −8.7 years. Larger age span and better prediction estimates of epigenetic aging possibly developed in the future may improve epigenetic clocks’ predictive power with respect to functional capacity. Some major strengths included the study’s longitudinal design and the fact that physical functioning was measured under standardized laboratory conditions using several tests. In future, after further development, epigenetic clocks (29) may be utilized to predict adverse health outcomes, perhaps to estimate the sensitivity of older patients to different therapies and follow intervention effects on aging. Further studies comprising longitudinal study settings are needed to clarify whether epigenetic aging explains the decline in physical functioning with aging or whether DNAm age simply illustrates the progress of aging.

In conclusion, our study suggests that current epigenetic clocks do not provide strong benefits over chronological age in predicting the development of future disability in old age. The epigenetic clock DNAm GrimAge was associated with decline in physical functioning over a 3-year follow-up in older women, while other epigenetic clocks were not associated with functional performance. However, larger studies with broader age range are needed to get a better picture about usability of epigenetic clocks in predicting age-related functional limitations and disabilities.

## Supplementary Material

glab270_suppl_Supplementary_Materials

## Data Availability

The twin data set used in the current study will be located in the Biobank of the National Institute for Health and Welfare, Finland. All the biobanked data are publicly available for use by qualified researchers following a standardized application procedure (https://thl.fi/en/web/thl-biobank/for-researchers).
