# Supplementary material for: The Association Between Epigenetic Clocks and Physical Functioning in Older Women: A 3-Year Follow-up
Source: J Gerontol A Biol Sci Med Sci. 2021 Sep 20;77(8):1569–76. doi: 10.1093/gerona/glab270 (PMC9373966; doi:10.1093/gerona/glab270)
Supplement: glab270_suppl_Supplementary_Materials [file glab270_suppl_supplementary_materials.pdf]

Supplementary material

Standardized laboratory measurements of physical functioning

During the walk tests, the participants wore walking shoes or sneakers, and use of walking aids that they would normally require was allowed. The Timed Up and Go (TUG) test was used to assess the participants' mobility, balance, and walking ability.[1-3] The participants were verbally and visually instructed to rise from a chair, walk three meters, turn around, walk back to the chair, and sit down. The test was done twice, and the faster performance was documented as the result. In the 10-meter walk test, three meters were allowed for acceleration, and walking time over 10 meters was recorded in the laboratory corridor using photocells for timing.[4, 5] The participants were instructed to walk as fast as possible without compromising their safety. The test was conducted twice, and the faster performance was documented as the result. Aerobic performance was assessed using a validated six-minute walk test.[6] The participants were instructed to walk as fast as possible up and down a straight 50-m indoor track for six minutes without compromising their safety. The distance (m) covered in that time was recorded.

Maximal isometric muscle strength measurements were performed on the dominant side, in a sitting position, using an adjustable dynamometer chair (Good Strength, Metitur, Palokka, Finland). *Grip strength* was measured with a dynamometer fixed to the arm of the chair, with the elbow flexed at 90°. *Ankle plantar flexion strength* measured the ankle at an angle of 90°, fastened by a belt to a strain-gauge system. The leg was elevated to a horizontal position, and the knee was set at an angle of 20° from full extension. *Knee extension strength* was measured at a knee angle of 60° from full extension, with the ankle fastened by a belt to a strain-gauge system. After familiarization, three to five maximal efforts, separated by a one-minute rest, were conducted. For each subject and strength test, the best performance with the highest value was used in the analysis.

Supplemental figure 1 shows the Bland-Altman plot for agreement between chronological age and Horvath´s DNAmAge. Average age measure difference was -1.71 years (std. err. 0.22, $P < 0.001$). The Spearman correlation between mean and difference of the measurements was 0.53, $P < 0.001$. The figure shows that greater discrepancies between chronological and Horvath´s DNAmAge are observed both for low and high measurement averages. For lower averages, Horvath´s DNAmAge is higher than chronological age leading to negative differences, while for higher average values Horvath´s DNAmAge is lower than chronological age (positive differences). The intraclass and concordance correlation coefficient estimates were moderate suggesting greater similarity between chronological and Horvath´s DNAmAge.

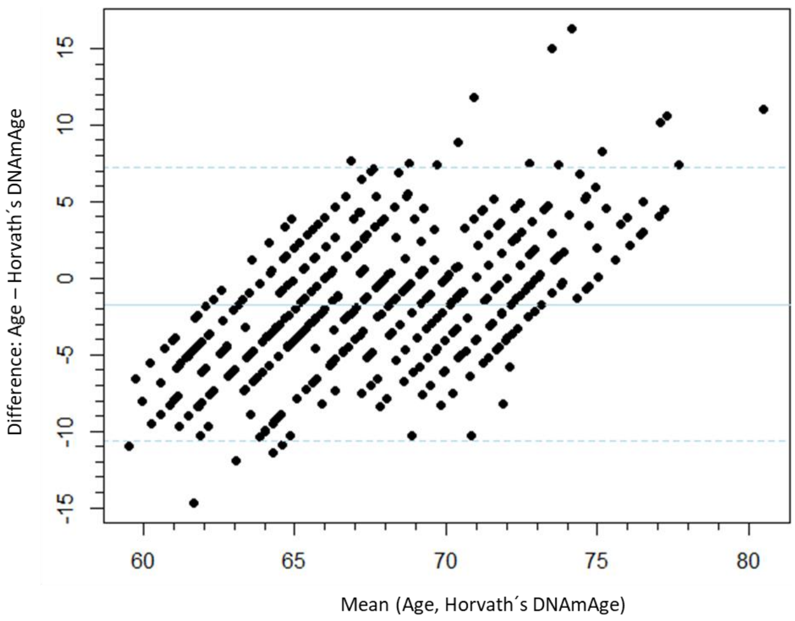

Supplement Figure 1. Bland-Altman plot for mean of chronological age and Horvath´s DNAmAge against difference between chronological age and Horvath´s DNAmAge (ICC = intraclass correlation coefficient for chronological age and Horvath´s DNAmAge, CCC = concordance correlation coefficient for chronological age and Horvath´s DNAmAge).

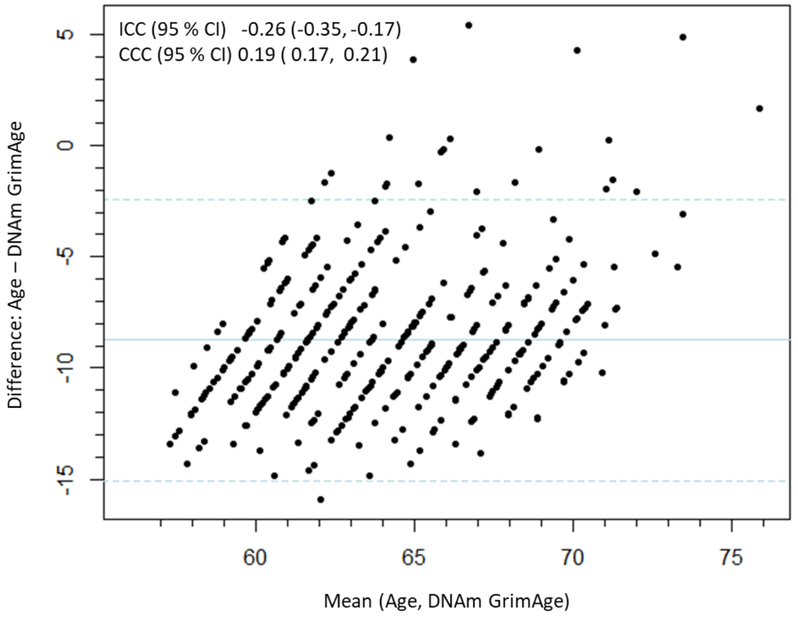

Supplement Figure 2. Bland-Altman plot for mean of chronological age and DNAm GrimAge against difference between chronological age and DNAm GrimAge (ICC = intraclass correlation coefficient for chronological age and DNAm GrimAge, CCC = concordance correlation coefficient for chronological age and DNAm GrimAge).

Supplemental figure 2 shows the Bland-Altman plot for agreement between chronological age and DNAm GrimAge. Average difference of the age measures was -8.75 years (std. err. 0.16, $P < 0.001$). The Spearman correlation between mean and difference of the measurements was 0.27, $P < 0.001$. The figure shows that greater discrepancies between chronological and DNAm GrimAge are observed for higher average measurements. For higher age averages chronological age was markedly higher than DNAm GrimAge. The intraclass and concordance correlation coefficient estimates were low suggesting greater average difference for the chronological and DNAm GrimAge measures.

Supplementary Table 1. Unadjusted regression coefficient estimates, standard errors and uncorrected p-values for linear associations between PhenoAge and Hannum age acceleration and chronological age and body mass index and physical functioning phenotypes at baseline (N = 413) and at three-year follow-up (N = 298) among older women.

**Outcome**	**AgeAccel**	**Time point**	**Multiplier**	**Est.**	**SE**	***p***
Body mass index (kg/m^2)	PhenoAge	baseline	--	0.072	0.036	**0.043**
		follow-up	--	0.060	0.039	0.124
	Hannum	baseline	--	0.050	0.053	0.344
		follow-up	--	0.070	0.054	0.198
	Age	baseline	--	0.003	0.082	0.975
		follow-up	--	0.043	0.092	0.641
Timed up and go (s)	PhenoAge	baseline	10	0.001	0.001	0.188
		follow-up	10	0.001	0.001	0.119
	Hannum	baseline	10	0.001	0.001	0.274
		follow-up	10	0.001	0.001	0.239
	Age	baseline	10	0.006	0.002	**0.001**
		follow-up	10	0.007	0.002	**<0.001**
10-meter walking test (s)	PhenoAge	baseline	--	0.000	0.000	0.412
		follow-up	--	0.001	0.000	0.120
	Hannum	baseline	--	0.000	0.000	0.364
		follow-up	--	0.001	0.001	0.073
	Age	baseline	--	0.003	0.001	**<0.001**
		follow-up	--	0.004	0.001	**<0.001**
Six-minute walking test (m)	PhenoAge	baseline	1 / 1 000	-0.001	0.001	0.368
		follow-up	1 / 1 000	-0.001	0.001	0.082
	Hannum	baseline	1 / 1 000	-0.001	0.001	0.371
		follow-up	1 / 1 000	-0.002	0.001	0.096
	Age	baseline	1 / 1 000	-0.007	0.002	**<0.001**
		follow-up	1 / 1 000	-0.010	0.002	**<0.001**
Grip strength (N)	PhenoAge	baseline	1 / 100	-0.007	0.006	0.182
		follow-up	1 / 100	-0.001	0.007	0.919
	Hannum	baseline	1 / 100	-0.002	0.007	0.793
		follow-up	1 / 100	0.013	0.007	0.051
	Age	baseline	1 / 100	-0.019	0.009	**0.044**
		follow-up	1 / 100	-0.039	0.012	**0.001**
Ankle plantar flexion strength (N)	PhenoAge	baseline	--	-0.016	0.038	0.662
		follow-up	--	-0.015	0.040	0.697
	Hannum	baseline	--	-0.054	0.056	0.339
		follow-up	--	-0.021	0.056	0.707
	Age	baseline	--	-0.199	0.076	**0.009**
		follow-up	--	-0.146	0.078	0.062

Knee extension strength (N)	PhenoAge	baseline	1 / 100	0.002	0.009	0.843
		follow-up	1 / 100	-0.003	0.009	0.714
	Hannum	baseline	1 / 100	0.000	0.014	0.975
		follow-up	1 / 100	-0.005	0.013	0.690
	Age	baseline	1 / 100	-0.070	0.017	**<0.001**
		follow-up	1 / 100	-0.062	0.016	**<0.001**

Note. Outcome values were scaled with the multiplier value. AgeAccel: DNA methylation age acceleration, Est: unadjusted regression coefficient, SE: standard error based on 10 000 bootstrap draws, *p*: expected false positive rate for a single test.

Supplementary Table 2. Adjusted regression coefficient estimates, standard errors and uncorrected p-values for linear associations between Grimage and Horvath age acceleration and chronological age and body mass index and physical functioning phenotypes at baseline (N = 413) and at three-year follow-up (N = 298) among older women.

				Adjusted for set 1			Adjusted for set 2		
**Outcome**	**AgeAccel**	**Time point**	**Multiplier**	**Est.**	**SE**	***p***	**Est.**	**SE**	***p***
Body mass index (kg/m^2)	GrimAge	baseline	--	0.088	0.086	0.305	0.065	0.086	0.447
		follow-up	--	0.083	0.091	0.358	0.058	0.090	0.518
	Horvath	baseline	--	0.103	0.049	**0.037**	0.102	0.050	**0.039**
		follow-up	--	0.087	0.053	0.096	0.088	0.053	0.098
	Age	baseline	--	-0.003	0.082	0.970	-0.024	0.084	0.777
		follow-up	--	0.036	0.092	0.692	0.017	0.095	0.855
Timed up and go (s)	GrimAge	baseline	10	0.005	0.002	**0.004**	0.004	0.002	**0.011**
		follow-up	10	0.006	0.002	**0.001**	0.005	0.002	**0.003**
	Horvath	baseline	10	0.000	0.001	0.764	0.001	0.001	0.549
		follow-up	10	0.000	0.001	0.995	0.000	0.001	0.777
	Age	baseline	10	0.006	0.002	**0.001**	0.004	0.002	**0.013**
		follow-up	10	0.007	0.002	**<0.001**	-0.001	0.006	0.893
10-meter walking test (s)	GrimAge	baseline	--	0.002	0.001	**0.024**	0.001	0.001	**0.050**
		follow-up	--	0.003	0.001	**<0.001**	0.003	0.001	**0.001**
	Horvath	baseline	--	-0.004	0.005	0.440	0.000	0.000	0.882
		follow-up	--	0.001	0.000	0.235	0.001	0.000	0.174
	Age	baseline	--	0.003	0.001	**<0.001**	0.002	0.001	**0.002**
		follow-up	--	0.004	0.001	**<0.001**	0.003	0.002	0.129
Six-minute walking test (m)	GrimAge	baseline	1 / 1 000	-0.005	0.002	**0.005**	-0.004	0.002	**0.010**
		follow-up	1 / 1 000	-0.007	0.002	**<0.001**	-0.006	0.002	**<0.001**
	Horvath	baseline	1 / 1 000	0.000	0.001	0.957	0.000	0.001	0.828

		follow-up	1 / 1 000	-0.001	0.001	0.362	-0.001	0.001	0.258
	Age	baseline	1 / 1 000	-0.007	0.002	**<0.001**	-0.006	0.003	**0.047**
		follow-up	1 / 1 000	-0.010	0.002	**<0.001**	-0.010	0.004	**0.013**
Grip strength (N)	GrimAge	baseline	1 / 100	-0.012	0.010	0.226	-0.009	0.009	0.331
		follow-up	1 / 100	-0.016	0.012	0.181	-0.012	0.012	0.317
	Horvath	baseline	1 / 100	-0.002	0.007	0.793	-0.002	0.006	0.736
		follow-up	1 / 100	0.013	0.007	0.051	0.013	0.007	0.061
	Age	baseline	1 / 100	-0.019	0.009	**0.042**	-0.016	0.009	0.087
		follow-up	1 / 100	-0.039	0.012	**0.001**	-0.036	0.012	**0.002**
Ankle plantar flexion strength (N)	GrimAge	baseline	--	-0.145	0.074	0.051	-0.135	0.074	0.068
		follow-up	--	-0.193	0.088	**0.028**	-0.168	0.087	0.052
	Horvath	baseline	--	-0.044	0.056	0.433	-0.039	0.055	0.477
		follow-up	--	0.037	0.059	0.537	0.036	0.059	0.548
	Age	baseline	--	-0.199	0.076	**0.009**	-0.195	0.150	0.194
		follow-up	--	-0.149	0.078	0.054	-0.235	0.260	0.366
Knee extension strength (N)	GrimAge	baseline	1 / 100	-0.033	0.019	0.076	-0.028	0.018	0.115
		follow-up	1 / 100	-0.066	0.019	**<0.001**	-0.056	0.018	**0.002**
	Horvath	baseline	1 / 100	0.024	0.013	0.075	0.017	0.012	0.155
		follow-up	1 / 100	0.012	0.012	0.319	0.005	0.011	0.619
	Age	baseline	1 / 100	-0.072	0.017	**<0.001**	-0.057	0.018	**0.001**
		follow-up	1 / 100	-0.063	0.016	**<0.001**	-0.048	0.036	0.183

Note. Covariate set 1 included smoking status, covariate set 2 included smoking status, alcohol consumption and number of chronic conditions. Outcome values were scaled with the multiplier value. Est: adjusted regression coefficient, SE: standard error based on 10 000 bootstrap draws, *p*: expected false positive rate for a single test.

Supplementary Table 3. Adjusted regression coefficient estimates, standard errors and uncorrected p-values for linear associations between PhenoAge and Hannum age acceleration and chronological age and body mass index and physical functioning phenotypes at baseline (N = 413) and at three-year follow-up (N = 298) among older women.

				Adjusted for set 1			Adjusted for set 2		
**Outcome**	**AgeAccel**	**Time point**	**Multiplier**	**Est.**	**SE**	***p***	**Est.**	**SE**	***p***
Body mass index (kg/m^2)	PhenoAge	baseline	--	0.071	0.036	**0.047**	0.068	0.035	0.054
		follow-up	--	0.059	0.039	0.129	0.054	0.038	0.154
	Hannum	baseline	--	0.050	0.053	0.350	0.040	0.053	0.454
		follow-up	--	0.070	0.054	0.201	0.060	0.055	0.275
	Age	baseline	--	-0.003	0.082	0.970	-0.024	0.084	0.777
		follow-up	--	0.036	0.092	0.692	0.017	0.095	0.855
Timed up and go (s)	PhenoAge	baseline	10	0.001	0.001	0.203	0.001	0.001	0.165
		follow-up	10	0.001	0.001	0.129	0.001	0.001	0.116
	Hannum	baseline	10	0.001	0.001	0.290	0.001	0.001	0.307
		follow-up	10	0.001	0.001	0.259	0.001	0.001	0.291
	Age	baseline	10	0.006	0.002	**0.001**	0.004	0.002	**0.013**
		follow-up	10	0.007	0.002	**<0.001**	-0.001	0.006	0.893
10-meter walking test (s)	PhenoAge	baseline	--	0.000	0.000	0.449	0.000	0.000	0.408
		follow-up	--	0.001	0.000	0.125	0.001	0.000	0.126
	Hannum	baseline	--	0.000	0.000	0.384	0.000	0.000	0.487
		follow-up	--	0.001	0.001	0.072	0.001	0.000	0.131
	Age	baseline	--	0.003	0.001	**<0.001**	0.002	0.001	**0.002**
		follow-up	--	0.004	0.001	**<0.001**	0.003	0.002	0.129
Six-minute walking test (m)	PhenoAge	baseline	1 / 1 000	-0.001	0.001	0.376	-0.001	0.001	0.408
		follow-up	1 / 1 000	-0.002	0.001	0.085	-0.001	0.001	0.082
	Hannum	baseline	1 / 1 000	-0.001	0.001	0.377	-0.001	0.001	0.569

		follow-up	1 / 1 000	-0.002	0.001	0.099	-0.002	0.001	0.165
	Age	baseline	1 / 1 000	-0.007	0.002	**<0.001**	-0.006	0.003	**0.047**
		follow-up	1 / 1 000	-0.010	0.002	**<0.001**	-0.010	0.004	**0.013**
Grip strength	PhenoAge	baseline	1 / 100	-0.006	0.005	0.171	-0.006	0.005	0.192
(N)		follow-up	1 / 100	0.005	0.005	0.314	0.007	0.005	0.184
	Hannum	baseline	1 / 100	-0.008	0.006	0.183	-0.007	0.006	0.230
		follow-up	1 / 100	-0.001	0.007	0.915	0.000	0.007	0.952
	Age	baseline	1 / 100	-0.019	0.009	**0.042**	-0.016	0.009	0.087
		follow-up	1 / 100	-0.039	0.012	**0.001**	-0.036	0.012	**0.002**
Ankle plantar	PhenoAge	baseline	--	-0.017	0.038	0.651	-0.018	0.037	0.629
flexion strength		follow-up	--	-0.014	0.039	0.715	-0.010	0.039	0.798
(N)	Hannum	baseline	--	-0.053	0.056	0.338	-0.042	0.054	0.436
		follow-up	--	-0.021	0.056	0.700	-0.018	0.055	0.741
	Age	baseline	--	-0.199	0.076	**0.009**	-0.195	0.150	0.194
		follow-up	--	-0.149	0.078	0.054	-0.235	0.260	0.366
Knee extension	PhenoAge	baseline	1 / 100	0.002	0.009	0.792	-0.001	0.008	0.928
strength (N)		follow-up	1 / 100	-0.003	0.009	0.748	-0.005	0.008	0.587
	Hannum	baseline	1 / 100	0.000	0.014	0.986	-0.003	0.012	0.834
		follow-up	1 / 100	-0.005	0.013	0.698	-0.007	0.012	0.561
	Age	baseline	1 / 100	-0.072	0.017	**<0.001**	-0.057	0.018	**0.001**
		follow-up	1 / 100	-0.063	0.016	**<0.001**	-0.048	0.036	0.183

Note. Covariate set 1 included smoking status, covariate set 2 included smoking status, alcohol consumption and number of chronic conditions. Outcome values were scaled with the multiplier value. Est: adjusted regression coefficient, SE: standard error based on 10 000 bootstrap draws, *p*: expected false positive rate for a single test.

Supplementary Table 4. Unadjusted estimates, standard errors, and uncorrected p-values for linear and quadratic effects of four age-acceleration measures and age on body mass index at baseline (N = 413) and at three-year follow-up (N = 298) among older women.

AgeAccel	Time point	Effect Component[a]	Unadjusted			Adjusted for set 1			Adjusted for set 2		
			Est.	SE	*p*	Est.	SE	*p*	Est.	SE	*p*
GrimAge	baseline	Linear	0.164	0.084	0.052	0.187	0.088	**0.034**	0.161	0.088	0.066
		Quadratic	-0.033	0.014	**0.018**	-0.029	0.014	**0.042**	-0.028	0.014	**0.041**
	follow-up	Linear	0.163	0.089	0.067	0.189	0.093	**0.042**	0.161	0.092	0.082
		Quadratic	-0.036	0.014	**0.009**	-0.032	0.014	**0.026**	-0.031	0.014	**0.026**
Horvath	baseline	Linear	0.109	0.051	**0.033**	0.114	0.052	**0.028**	0.111	0.052	**0.033**
		Quadratic	-0.005	0.007	0.449	-0.005	0.007	0.446	-0.004	0.007	0.542
	follow-up	Linear	0.093	0.055	0.091	0.098	0.055	0.076	0.096	0.055	0.083
		Quadratic	-0.004	0.007	0.561	-0.005	0.007	0.545	-0.003	0.008	0.650
PhenoAge	baseline	Linear	0.087	0.038	**0.022**	0.085	0.037	**0.023**	0.083	0.037	**0.028**
		Quadratic	-0.008	0.004	**0.036**	-0.008	0.004	**0.040**	-0.009	0.004	**0.029**
	follow-up	Linear	0.075	0.041	0.070	0.073	0.041	0.073	0.069	0.040	0.089
		Quadratic	-0.008	0.008	0.072	-0.008	0.004	0.081	-0.008	0.004	0.063
Hannum	baseline	Linear	0.056	0.053	0.288	0.056	0.053	0.294	0.047	0.053	0.376
		Quadratic	-0.006	0.008	0.459	-0.006	0.008	0.475	-0.008	0.008	0.337
	follow-up	Linear	0.076	0.055	0.167	0.075	0.055	0.170	0.067	0.055	0.228
		Quadratic	-0.008	0.008	0.367	-0.007	0.008	0.378	-0.009	0.008	0.274
Age[b]	baseline	Linear	-0.009	0.086	0.922	0.002	0.086	0.981	-0.016	0.087	0.855
		Quadratic	-0.011	0.027	0.702	-0.009	0.027	0.744	-0.014	0.028	0.612
	follow-up	Linear	0.049	0.096	0.606	0.042	0.095	0.662	0.025	0.097	0.793
		Quadratic	-0.012	0.031	0.706	-0.010	0.031	0.746	-0.015	0.031	0.641

Note. [a]Curvilinear age-acceleration and age associations modelled using second-degree polynomial: $h + h^2$, where h is the linear component and h^2 is the quadratic component. [b]Age was centered. Covariate set 1 included smoking status, covariate set 2 included smoking status, alcohol consumption and number of chronic conditions. Est: adjusted regression coefficient, SE: standard error based on 10 000 bootstrap draws, *p*: expected false positive rate for a single test.

REFERENCES

1. Podsiadlo D and Richardson S. "The timed 'Up & Go': A test of basic functional mobility for frail elderly persons". J Am Geriatr Soc 1991; 39(2): 142-148.

2. Collado-Mateo D, Domínguez-Muñoz F, Adsuar J, et al. Reliability of the Timed Up and Go Test in Fibromyalgia. Rehabil Nur 2018; 43(1): 35-39.

3. Hafsteinsdóttir TB, Rensink M and Schuurmans M. Clinimetric properties of the Timed Up and Go Test for patients with stroke: a systematic review. Top Stroke Rehabil 2014; 21(3): 197-210.

4. Tiainen K, Pajala S, Sipilä S, et al. Genetic effects in common on maximal walking speed and muscle performance in older women. Scand J Med Sci Sports 2007; 17(3): 274-280.

5. Sipilä S, Multanen J, Kallinen M, et al. Effects of strength and endurance training on isometric muscle strength and walking speed in elderly women. Acta Physiol Scand 1996; 156(4): 457-464.

6. Aniansson A, Rundgren A and Sperling L. Evaluation of functional capacity in activities of daily living in 70-year-old men and women. Scand J Rehabil Med 1980; 12(4): 145-154.
